# Assessment of the Risk of Type 2 Diabetes Mellitus Among a Rural Population in South India Using the Indian Diabetic Risk Score

**DOI:** 10.7759/cureus.41880

**Published:** 2023-07-14

**Authors:** Angeline Jeyaseeli V, Ganesan R, Dhibika Mathivanan, Allen Prabagaran P

**Affiliations:** 1 Physiology, Sri Venkateshwaraa Medical College Hospital and Research Centre, Puducherry, IND; 2 Microbiology, Indira Gandhi Medical College & Research Institute, Puducherry, IND; 3 Orthopedics, Christian Medical College, Vellore, IND

**Keywords:** bmi, family history of diabetes, type 2 diabetes mellitus, risk assessment, indian diabetic risk score

## Abstract

Objective

The objective of our study was to assess the risk for diabetes using the Indian Diabetic Risk Score (IDRS) questionnaire and compare the components of IDRS between the risk groups.

Methods

It was a cross-sectional study involving 270 male and female attendees who visited Melmaruvathur Adhiparasakthi Institute of Medical Sciences (MAPIMS) from December 2019 to May 2020. The diabetes risk was assessed using the IDRS questionnaire. Statistical Package for Social Sciences (SPSS) version 20 (IBM Corp., Armonk, NY) was used for statistical analysis. *P* < 0.05 was considered statistically significant.

Results

IDRS categorization showed 12.6%, 73.7%, and 13.7% in the low-risk, moderate-risk, and high-risk groups, respectively. Age, waist circumference, and body mass index (BMI) were significantly (*P *< 0.05) higher in the high-risk group when compared with the low-risk group. Subjects with a positive family history of diabetes and no/mild physical activity were higher in the moderate and high-risk group but there is no significant association present between them.

Conclusion

The current study estimates the effectiveness of IDRS in identifying people at high risk for diabetes in the community. This study also emphasizes the need for early identification of high-risk individuals and planning for the appropriate intervention to prevent, or delay, the onset of diabetes and thus reduces the burden of diabetes in India.

## Introduction

Type 2 diabetes mellitus (T2DM) is a non-communicable disease (NCD) that has shown a maximum increase in India during the last few years. According to recent reports, India ranks second in the world with a diabetes population of 69.2 million and may have 123.5 million diabetics by 2040 [1]. It is important to identify individuals with undiagnosed T2DM to prevent or delay T2DM complications [2]. Mohan et al. designed and developed the Indian Diabetes Risk Score (IDRS), a simple and effective screening tool to identify individuals who are at risk of developing diabetes in the future. It uses four parameters to calculate the score: age, family history of diabetes in parents, waist circumference, and physical activity. This questionnaire has a maximum score of 100; calculated by combining the scores of all the above parameters. Based on the IDRS scores, subjects were labeled as low risk (IDRS < 30); moderate risk (IDRS 30-50), and high risk of diabetes (IDRS > 60) [3]. The aim of our study was to evaluate the risk of diabetes using the IDRS questionnaire and to compare the components of the IDRS between risk groups.

## Materials and methods

Study design

The study was conducted in the Department of Physiology, Melmaruvathur Adhiparasakthi Institute of Medical Sciences and Research Institute (MAPIMS) from December 2019 to May 2020 after obtaining approval from the Institute Ethics Committee (Human Studies) of MAPIMS with registration number 126 (11) 2019. It was a cross-sectional study that involved 270 male and female attendees who visited MAPIMS Medical College and Hospitals during the study period. The sample size was estimated assuming that 40% of people would have a moderate to high risk score [4]. The sample size was estimated using the formula 4 pq/E², where prevalence (p) = 40%, q = 60%, and relative error (E) = 15% of prevalence, and the estimated sample size came out to be 266. In our study, we included 270 participants.

Selection of subjects

Subjects aged between 18 to 50 years who visited MAPIMS Medical College and Hospitals along with patients were included in the study. They were divided into three groups based on the Indian Diabetic Risk Score (IDRS:) GROUP I: Subjects with low risk (IDRS of < 30); GROUP II: Subjects with moderate risk (IDRS of 30-50); GROUP III: Subjects with high risk (IDRS of > 60) [3]. Subjects with diagnosed diabetes mellitus, hypertension, thyroid disorders, coronary disease, and other systemic illnesses (according to medical history) were excluded from the study.

Experimental design

Each study participant was assessed by the IDRS questionnaire, which uses age, abdominal obesity, family history of diabetes, and physical activity to assess the diabetic risk [3]. Study participants <35 years were marked as 0; 35-49 years as 1; ≥50 years as 2 and scores 0, 20, and 30 were given, respectively. Waist circumference was used for assessing abdominal obesity. It was measured from the circumference of the abdomen, at its narrowest point between the lower edge of the ribs (10th rib) and the top of the iliac crest. Female subjects with waist circumference <80 cm were marked as 0; ≥81-89 cm as 1; ≥90 cm as 2, and scores 0, 10, and 20 were given, respectively. Male subjects with waist circumference <90 cm were marked as 0; ≥91-99 cm as 1; ≥100 cm as 2, and scores 0, 10, and 20 were given, respectively. Physical activities were marked as 0 - for subjects performing regular exercise and performing strenuous (manual) activities at home/work; marked as 1 - for subjects performing regular exercise or performing strenuous (manual) activities at home/work; 2 - for subjects doing no exercise and/or sedentary activities at home/work, and the scores 0, 20, and 30 were given, respectively. Strenuous activities were defined as activities that cause an increased amount of exertion, fast breathing, and a significant increase in heart rate for at least 10 minutes continuously. Subjects were marked as 0 if there is no family history of diabetes; subjects were marked as 1 if one parent is diabetic, subjects were marked as 2 if both parents are diabetic, and scores 0, 10, and 20 were given, respectively. They were then divided into groups I, II, and III based on the IDRS score. Height (in cm) and weight (in kg) were recorded in all three groups. Body mass index (BMI) was then calculated using the Quetelet index = weight (kg)/(height)^2^ (m).

Statistical analysis of data

Statistical Package for the Social Sciences (SPSS) version 20 (IBM Corp., Armonk, NY) was used for statistical analysis. Continuous data such as age, BMI, and waist circumference were expressed as mean with standard deviation, and the intergroup differences in means were compared using a one-way analysis of variance (ANOVA) test. Qualitative data, such as physical activity and a family history of diabetes, were expressed as percentages, and the chi-square test (χ2) was used to determine the association between variables. “P” < 0.05 was considered statistically significant.

## Results

Group-wise distribution of subjects

Based on the IDRS score, participants were grouped into low risk (GROUP I), moderate risk (GROUP II), and high risk (GROUP III) as shown in Table 1. Out of the total 270 participants, the total number of male participants was 146 (54%), of which 19 participants (13%) were in the low-risk group; 109 participants (74.6%) were in the moderate-risk group; 18 (12.3%) were in the high-risk group. The total number of female participants was 124 (46%), of which 15 participants (12%) were in the low-risk group; 90 participants (72.5%) came in the moderate-risk group; 19 participants (15.3%) came in the high-risk group.

**Table 1:** Group‑wise distribution of subjects in risk groups (n=270) Values are expressed as frequency (percentage).

Group (risk score)	No. of subjects Percentage	Males	Females
GROUP I (<30) - Low risk	34 (12.6 %)	19 (13 %)	15 (12 %)
GROUP II (30‑50) - moderate risk	199 (73.7 %)	109 (74.6 %)	90 (72.5 %)
GROUP III (≥60) - High risk	37 (13.7 %)	18 (12.3 %)	19 (15.3 %)
TOTAL	270 (100 %)	146 (54 %)	124 (46 %)

Distribution of Indian Diabetic Score components

IDRS utilizes four parameters to assess an individual’s diabetic risk; each parameter has different subsets with different scores. Table 2 shows the overall distribution of study participants within each subset of the IDRS components.

**Table 2:** Indian Diabetic Score distribution (n=270) IDRS = Indian Diabetes Risk Score

IDRS components	Number of subjects	Percentage
Age (years)		
< 35	130	48.1%
35 - 49	117	43.3%
≥ 50	23	8.5%
Waist circumference (cm)		
Waist <80 cm (female), <90 (male)	63	23.3%
Waist ≥ 80 – 89 cm (female), ≥ 90 – 99 cm (male)	146	54.07%
Waist ≥90 cm (female), ≥ 100 cm (male)	61	22.6%
Physical activity		
Exercise (regular) + strenuous work	49	18.1%
Exercise (regular) or strenuous work	197	73.0%
No exercise and sedentary work	24	8.9%
Family history of diabetes		
No diabetes in parents	221	81.9%
One parent is diabetic	37	13.7%
Both parents are diabetic	12	4.4%

Gender‑wise distribution of waist circumference of subjects in risk groups

Waist circumference was used as a marker for grading abdominal obesity. IDRS uses different waist circumference cutoff values for males and females. The mean waist circumference for the males and females of our study group was given in Table 3. Comparison of waist circumference among the groups was done using one-way ANOVA and the Tukey post-hoc test was performed to find the significant difference among the group.

**Table 3:** Gender‑wise distribution of the waist circumference of subjects in risk groups (n=270) Values are expressed as mean/Standard deviation (SD); Comparison of variables between groups done using ANOVA, *p<0.05 is statistically significant among groups.

Waist circumference	Total (n=270)	Low risk (n=34)	Moderate risk (n=199)	High risk (n=37)	P value*
Male	96.24±14.2	84.36 ± 9.32	93.42±7.06	99.47±14.21	0.002
Female	89.00±8.39	83.6 ±11.23	88.23±5.04	93.56±12.98	0.004

Distribution of family history of diabetes and physical activity of subjects in different risk groups

The association of positive family history of diabetes and less physical activity with the moderate and high-risk groups was done using the chi-square test as shown in Table 4.

**Table 4:** Distribution of the family history of diabetes and physical activity among subjects in risk groups (n=270) Values are expressed as frequency (percentage); the comparison of variables between groups was done using the chi-square test; *p<0.05 is statistically significant among groups

Variables	Total (%) (n=270)	Low risk (%) (n=34)	Moderate & high risk (%) (n=236)	P value*
Family History of Diabetes				
Present	49 (18.1 %)	7 (14.3 %)	42 (85.8 %)	0.883
Absent	221 (81.9 %)	27 (12.21 %)	194 (87.74 %)	
Physical activity %				
Exercise / strenuous work	246 (91.11 %)	30 (12.19 %)	217 (88.2 %)	0.544
No exercise / strenuous work	24 (8.9 %)	4 (16.6 %)	20 (80.33 %)	

Distribution of age and body mass index (BMI) among subjects of subjects in different risk groups

Comparison of mean age and BMI between the groups was done using one-way ANOVA and the Tukey post-hoc test was performed to find the significant difference among the group as shown in Table 5.

**Table 5:** Distribution of age and BMI among subjects in risk groups (n=270) Values are expressed as mean/standard deviation (SD); comparison of variables between groups done using ANOVA, *p<0.05 is statistically significant among groups; BMI = body mass index; ANOVA = analysis of variance

Variables	Total (n=270)	Low risk (%) (n=34)	Moderate risk (%) (n=199)	High risk (%) (n=37)	P value*
Age	36.91±9.87	34.35±1.26	41.52±3.04	44.2±11.08	0.009
BMI	23.15±3.47	21.42±9.6	24.47±6.02	27.36±7.04	0.005

## Discussion

Of the total of 270 participants, 34 participants (12.6%) were in the low-risk group; 199 participants (73.7%) were in the medium-risk group; 37 participants (13.7%) were in the high-risk group according to the IDRS. Similar findings for the high-risk category were reported in studies conducted by Subramani et al. (12.1%) and Kumar et al. (18.6%) [5,6]. Low values ​​for high-risk categories were observed in studies by Singh MM et al. (1%), Gopalakrishnan et al. (1.9%), and Bhatia et al. (1%) [7-9]. These studies were conducted on medical students so they would have reduced scores for IDRS components such as age, physical activity, and waist circumference. High values ​​(31.2% and 31.5%) for the high-risk category were shown by studies conducted by Mohan et al. and Chowdhury et al. [10,11]. In both studies, participants over the age of 50 were present in a higher proportion than in our study. This would affect the IDRS component scores and increase the number of people in the high-risk category. A study conducted by Bhatia et al. (68%) and Subramani et al. (74.7%) [5-9] showed similar results for the intermediate risk category while in the studies conducted by Chowdhury et al. (46%), Mohan et al. (50.3%), Vardhan et al. (28%), and Singh MM et al. (22%) [7,10-12] reported lower values ​​for the intermediate risk category. In these studies, the differences in study population characteristics may have influenced the IDRS. Of the 270 study participants, 49 (18.1%) had a positive family history of diabetes. Study participants with a positive family history of diabetes were present in a high percentage in the moderate and high-risk groups (85.8%). These findings were consistent with the findings of previous studies that family history is an independent risk factor for type 2 diabetes mellitus (T2DM) [13]. However, in our study, we were unable to demonstrate any significant association between a positive family history of diabetes and a moderate to high risk of diabetes. Studies conducted by Subramani et al. (16.6%), Bhatia et al. (32%), Gopalakrishnan et al. (46.6%), and Singh MM et al. (41.5%) [5,7-9] showed a significant association between a positive family history of diabetes and moderate to high risk of diabetes. Studies with a large sample size than our study were done to reveal the association between a positive family history of diabetes and a moderate to high risk of diabetes. In our study, 49 (18.1%) subjects regularly exercised and did strenuous activities, 197 (73%) subjects regularly exercised or did strenuous activities, and 24 (8.9%) subjects did not exercise. Subjects with no or less physical activity/exercise were highly represented in the medium and high-risk groups (80.33%). Our study confirms previous studies showing that less physical activity increases the risk of T2DM [14]. However, no significant association was found between no/moderate physical activity and moderate to high risk of diabetes. This may be attributed to the sample size used in our study. Future studies done in larger populations could unveil the association between no/moderate physical activity and moderate to high risk of diabetes. In our study, the mean age of the subjects was 36.91±9.87. The mean age of the high-risk group (44.2±11.08) showed a statistically significant difference (P<0.009) compared to the low-risk group (34.35±1.26). This may be due to the fact that at older ages, people would have less physical activity and a higher waist circumference, thus increasing the IDRS. The average body mass index (BMI) of our study group was 23.15±3.47. Statistically significant (P<0.005) BMI values ​​were noted in the high-risk group compared to the low-risk group. These findings were consistent with previous studies showing that people with a higher BMI tend to develop T2DM at a younger age [15]. Similar observations were present in studies conducted by Gopalakrishnan et al., Singh MM et al., Kumar et al., and Chowdhury et al. [6-8,11]. The average waist circumference for men was 96.24±14.2 and for women was 89.00±8.39. Waist circumference in men and women from the high-risk group was significantly higher (P<0.002) and (P<0.004) as compared to the low-risk group. Waist circumference is a marker of central obesity, which can lead to cardiometabolic diseases [16]. Persons in the high-risk group had a higher waist circumference and are therefore at risk of developing cardiometabolic diseases. There is a paucity of literature revealing the use of the IDRS questionnaire as a tool to screen for diabetic risk in a South Indian rural population. Hence in our study, we assessed the diabetic risk using the IDRS questionnaire and compared the components of the IDRS between risk groups.

Limitations

The limitations of the present study were age group restrictions since the present study was conducted on male and female attendees who accompanied the patients between the age group of 18 and 50 years. Subjects aged more than 50 years were excluded from the study, considering that most of them will be diagnosed with T2DM or other comorbidities.

## Conclusions

As evident from our study, 199 participants (73.7%) came under the moderate-risk group; 37 participants (13.7%) came under the high-risk group. Age, waist circumference, and body mass index (BMI) were significantly (P < 0.05) higher in the high-risk group when compared with the low-risk group. Subjects with a positive family history of diabetes and no/mild physical activity were higher in the moderate and high-risk group but there is no significant association present between them. The current study estimates the effectiveness of the Indian Diabetic Risk Score (IDRS) for identifying people at high risk for diabetes in the community.
